# Normalized circulating Tfh and Th17 associates with improvement in myasthenia gravis treated with ofatumumab

**DOI:** 10.3389/fimmu.2024.1280029

**Published:** 2024-02-13

**Authors:** Xiaodong Song, Yang He, Yang Huo, Hong Jiang, Yao Yu, Yue Sun, Zunjing Liu, Zhaoxu Zhang

**Affiliations:** ^1^ Department of Neurology, Peking University People’s Hospital, Beijing, China; ^2^ Department of Neurology, The First Affiliated Hospital of Chongqing Medical University, Chongqing, China

**Keywords:** T follicular helper cells, T helper 17 cells, myasthenia gravis, ofatumumab, B cell depletion therapy

## Abstract

**Objective:**

To assess the effect of B cell depletion therapy (BCDT) on circulating T follicular helper (cTfh) and circulating T helper 17 (cTh17) cells and its relation to clinical improvement in patients with myasthenia gravis (MG).

**Methods:**

28 anti-AchR positive MG patients treated with ofatumumab and 28 healthy controls (HCs) were included. Frequencies of cTfh and cTh17 cells were monitored by flow cytometry at baseline and 4, and 12 weeks after the initial dose ofatumumab. Serum cytokines associated with cTfh and cTh17, including IL-6, IL-21, and IL-17, were also analyzed.

**Results:**

The frequency of cTfh and cTh17 significantly increased in MG patients compared with HCs. Additionally, elevated levels of both T-cell subsets correlated with MG severity. During the follow-up, cTfh and cTh17 return to normal after BCDT. Furthermore, the decrease in cTfh and cTh17 was associated with MG scores improvement over time. Notably, cTfh- and cTh17-related cytokines, including IL-6, IL-21, and IL-17, exhibited a marked decrease following ofatumumab therapy.

**Conclusions:**

Abnormal expansion of cTfh and cTh17 cells may be key features in the immunopathology of MG. Their levels returned to normal after BCDT, which was closely correlated with clinical amelioration. This result suggests that these two T-cell subsets may be targets for BCDT treatment of MG.

## 1 Introduction

Myasthenia gravis (MG) is a rare autoimmune disease characterized by fluctuating muscle weakness and fatigue (1). Autoantibodies against postsynaptic membrane components of the neuromuscular junction (NMJ) are present in most patients with MG, with acetylcholine receptor (AChR) antibodies being the most common (1). B-cell depletion therapy (BCDT) with rituximab has been successfully employed in treating generalized MG based on two recent randomized placebo-controlled clinical trials (2, 3). However, rituximab does not impair the production of bone marrow B cells or antibodies secreted by long-lived plasma cells (4). Moreover, the anti-AchR antibody tiers are not always correlated with the clinical severity (5, 6). Therefore, it is unlikely that disease remissions achieved through BCDT are solely a result of autoantibody depletion.

Increasing evidence showed that rituximab depletes B cells that could potentially interact with T cell subsets involved in autoimmune pathology (antibody-mediated autoimmune). For example, T follicular helper (Tfh) cells are a T cell subset distributed in the germinal center of secondary lymphoid tissues, which plays a central role in promoting B-cell maturation and antibody production (7). Patients with neuromyelitis optica spectrum disorder exhibit a disturbed circulating Tfh (cTfh) cell subset equilibrium that promotes B-cell differentiation (8). However, this imbalance can be rectified by treatment with rituximab (8). T helper (Th) 17 cells represent a distinct subgroup of CD4^+^ T helper cells known for their ability to produce the signature cytokine IL-17 (9). Research findings have consistently linked Th17 cells to tissue-specific autoimmune inflammatory disorders (9). BCDT was reported to decrease circulating Th17 (cTh17) cells in patients with primary Sjögren syndrome (10). Moreover, reduced levels of cTh17 cells and IL-17 are associated with disease amelioration and declined autoantibody titers (10).

In this study, we quantified the frequencies of cTfh and cTh17 in MG patients on ofatumumab monotherapies. Given that ofatumumab is a fast-acting B-cell depletion agent (11, 12), we hypothesized that the two T-cell subsets would change obviously after applying ofatumumab. Furthermore, we analyzed the relationship between the change in frequency cTfh and cTh17 and clinical improvement in patients with MG after treatment.

## 2 Materials and methods

### 2.1 Patients

We retrospectively included 28 anti-AchR positive MG patients attending Peking University People’s Hospital and 28 age-matched healthy controls (HCs) (**Table 1**). None of the HCs had a history of disease or received any treatment within the previous 6 months. Detailed past medical history and duration of follow-up for each MG patient was shown in **Supplementary Table 1**. In MG patients, the administration of ofatumumab involves a subcutaneous dose of 20 mg every 4 weeks following loading doses of 20 mg on days 1, 7, and 14. After starting ofatumumab monotherapy, all participants discontinued their previous immunosuppressive medications. Peripheral whole-blood samples were obtained at baseline, 4 weeks, and 12 weeks after the initial dose for antibody, cytokines, and flow cytometric analysis. At the same time, the clinical severity was evaluated with the Myasthenia Gravis Foundation of America quantitative myasthenia gravis (MGFA-QMG) score, the 15-item Myasthenia Gravis Quality of Life scale (MG-QOL15), and the MG-Related Activities of Daily Living (MG-ADL) score.

**Table 1:** Demographic characteristics of MG patients and HCs.

Characteristics	MG (n=28)	HC (n=28)	p-value
Age (years), mean (SD)	54.0 (13.4)	52.0 (15.3)	0.664
Female, n (%)	12 (42.9)	14 (50.0)	0.592
Disease duration (years), median (IQR)	1.5 (1.2-2.6)	–	–
Thymoma (thymectomy), n (%)	10 (35.7)	–	–
AChR antibody(nmol/L), median (IQR)	18.5 (13.4-26.8)	–	–
MGFA type, n (%)			
IIa	8 (28.6)	–	–
IIb	7 (25.0)	–	–
IIIa	6 (21.4)	–	–
IIIb	3 (10.7)	–	–
IVa	1 (3.6)	–	–
IVb	3 (10.7)	–	–
MGFA-QMG score, median (IQR)	14.5 (8.0-18.0)	–	–
MG-QOL15 score, median (IQR)	14.0 (8.0-17.3)	–	–
MG-ADL score, median (IQR)	8.0 (6.0-12.0)	–	–

MG, myasthenia gravis; HC, healthy control; SD, standard deviation; IQR, interquartile range; AChR, acetylcholine receptor; MGFA, Myasthenia Gravis Foundation of America; MGFA-QMG, Myasthenia Gravis Foundation of America quantitative myasthenia gravis score; MG-QOL15, the 15-item Myasthenia Gravis Quality of Life scale; MG-ADL, the MG-Related Activities of Daily Living score.

### 2.2 Flow cytometric analysis

**Figure 1:**
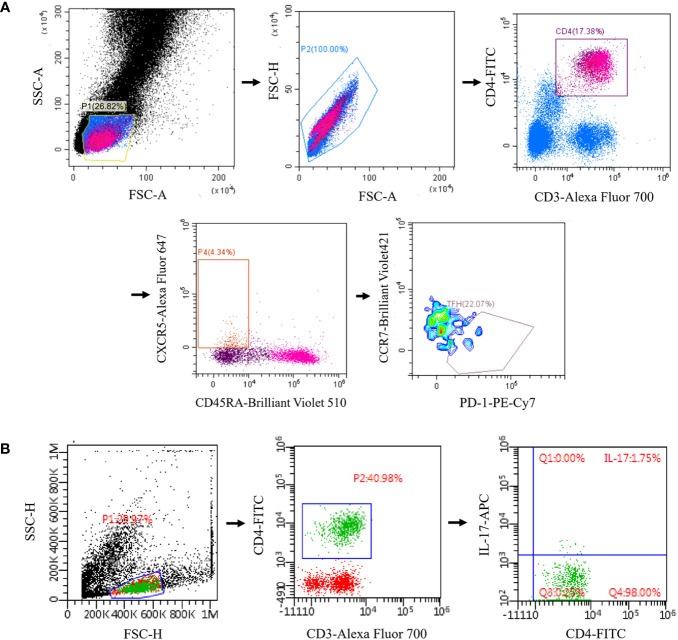
Reprehensive gating strategy for circulating Tfh and Tfh17 cells. **(A)** Tfh was marked as the CD3^+^ CD4^+^ CXCR5^+^ CD45RA^-^ CCR7^low^ PD-1^+^ lymphocytes; **(B)** Th17 was identified as CD3^+^ CD4^+^ IL17^+^ lymphocytes. Tfh, T follicular helper; Th17, T helper 17.

Trained technicians follow a standardized protocol for cryopreservation of whole blood samples within 2 hours of collection (13, 14). Briefly, the blood sample is mixed with a 20% DMSO solution (Beyotime) and then diluted 1:1 with RPMI medium (Gibco) (final concentration of 10% DMSO and 50% RPMI). The mixture was placed in a freezing tube and stored in liquid nitrogen. Peripheral blood mononuclear cells (PBMC) were isolated from whole blood by Ficoll-Hypaque density gradient centrifugation. The cells were cultured in RPMI 1640 medium and stimulated with phorbol 12-myristate 13-acetate (50 ng/ml, sigma-aldrich) plus ionomycin (500 ng/ml, BD Pharmingen) for 5 h in the presence of Brefeldin A (BD Pharmingen, 10 ug/ml). Cell surface markers were first stained with CD3-Alexa Fluor 700 (Biolegend), CD4-FITC (Biolegend), CD45RA-Brilliant Violet 510 (Biolegend), CXCR5-Alexa Fluor 647 (BD Biosciences), PD-1-PE-Cy7 (Biolegend), CCR7- Brilliant Violet 421 (Biolegend), CD25 (BD Biosciences), and CD127 (Biolegend). Then, the PBMCs were fixed and permeabilized by Foxp3/Transcription Factor Staining Buffer Set (eBioscience) and stained intracellularly with IL-17-APC (Biolegend). The frequencies of Tfh and Th17 cells of MG patients and HCs were assessed by flow cytometry using FACSAria III (BD Biosciences) and FlowJo software (Tree Star). cTfh was marked as the CD3^+^ CD4^+^ CXCR5^+^ CD45RA^-^ CCR7^low^ PD-1^+^ lymphocytes (15) (**Figure 1A**). cTh17 was identified as CD3^+^ CD4^+^ IL17^+^ lymphocytes (16) (**Figure 1B**). Regulatory T (Treg) cells was marked as the CD3^+^CD4^+^CD25^high^CD127^low^ lymphocytes (**Supplementary Figure 1**). All the three T cell types were assayed with the same flow cytometry panel.

### 2.3 Measurement of IL-6, IL-17, IL-21, AChR antibody in serum

The serum was rapidly separated from whole blood by centrifugation at 10,000g for 10 minutes and stored at -80°C. Serum levels of IL-6, IL-17, and IL-21 were determined by an enzyme-linked immunosorbent assay (ELISA) kit (eBioscience) according to the manufacturer’s instructions. Serum AChR autoantibody titers were measured using an ELISA kit from RSR Ltd, with positivity defined as ≥ 0.45 nmol/L.

### 2.4 Statistical analyses

Continuous variables are presented as mean [standard deviation (SD)] unless otherwise specified. Categorical variables were shown as numbers (percentages). Mann-Whitney U test was used for analyzing unpaired continuous data, and Pearson’s chi-square for categorical data. Wilcoxon signed-rank test was used to assess the difference between before and after ofatumumab treatment. Spearman rank correlation was applied for correlation analysis. A p-value < 0.05 indicated statistical significance. All data were analyzed with IBM SPSS version 22.0 (IBM Corp, Armonk, New York) and GraphPad Prism 9.5 (GraphPad Software Inc., La Jolla CA, USA).

## 3 Results

### 3.1 Increased cTfh and cTh17 correlate with MG severity

**Figure 2:**
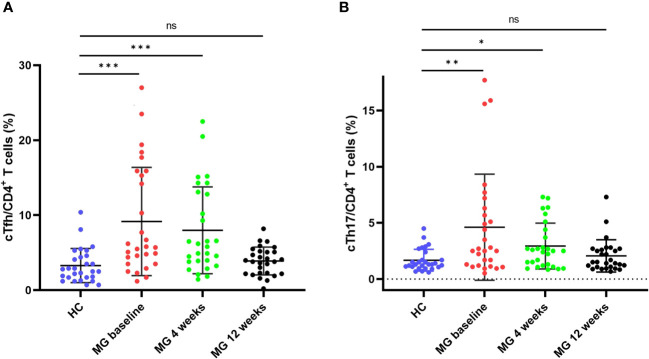
cTfh and cTh17 cell frequencies among CD4^+^ T cells in patients with MG and HCs. **(A)** cTfh cell frequency among CD4^+^ T cells in patients with MG and HCs; **(B)** cTh17 cell frequency among CD4^+^ T cells in patients with MG and HCs. cTfh, circulating T follicular helper; cTh17, circulating T helper 17;MG, myasthenia gravis; HCs, healthy controls. "ns", "*", "**", and "***" represent not significant, P < 0.05, P < 0.01, and P < 0.001, respectively.

**Figure 3:**
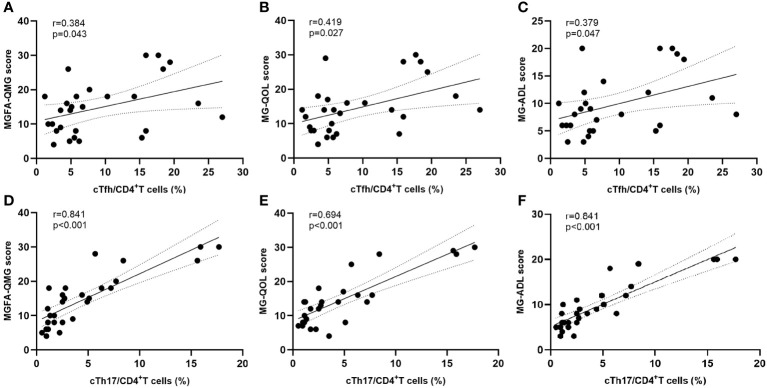
Correlation between cTfh and cTh17 cell frequency and clinical severity. **(A–C)** Association between circulating Tfh cell frequency and clinical severity at baseline; **(D–F)** Correlation between circulating Th17 cell frequency and clinical severity at baseline. cTfh, circulating T follicular helper; cTh17, circulating T helper 17; MGFA-QMG, Myasthenia Gravis Foundation of America quantitative myasthenia gravis score; MG-QOL15, the 15-item Myasthenia Gravis Quality of Life scale; MG-ADL, the MG-Related Activities of Daily Living score.

The frequencies of cTfh (9.2% vs 3.3%, p < 0.001) and cTh17 (4.6% vs 1.7%, p = 0.005) among CD4^+^ T cells were significantly higher in MG patients than in HCs (**Figures 2A, B**). No significant differences were observed in cTfh and cTh17 levels between MG patients with and without a history of thymoma (**Supplementary Table 2**). Additionally, there was a positive but moderate correlation between the frequency of cTfh and the MG scales (MGFA-QMG: r=0.0.384, p=0.043; MG-QOL15: r=0.419, p=0.027; MG-ADL: r=0.379, p=0.047) (**Figures 3A–C**). Also, a positive correlation was found between the percentages of cTh17 cells and the clinical severity score in MG patients at baseline (MGFA-QMG: r=0.841, p<0.001; MG-QOL15: r=0.694, p<0.001; MG-ADL: r=0.841, p<0.001) (**Figures 3D–F**).

### 3.2 Clinical improvement after ofatumumab treatment

**Figure 4:**
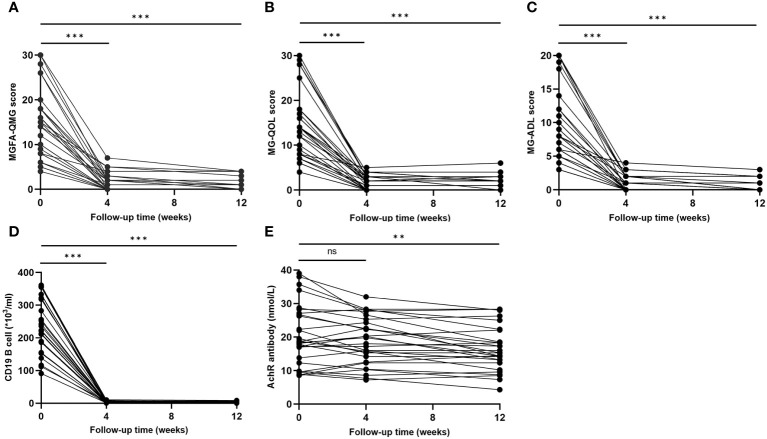
Clinical improvement after ofatumumab treatment. **(A–C)** MGFA-QMG, MG-QOL15, and MG-ADL scores significantly decreased at each visit; **(D, E)** Change of circulating CD19^+^ B and AChR antibody levels during the follow-up. MGFA-QMG, Myasthenia Gravis Foundation of America quantitative myasthenia gravis score; MG-QOL15, the 15-item Myasthenia Gravis Quality of Life scale; MG-ADL, the MG-Related Activities of Daily Living score; AChR, acetylcholine receptor. "ns", "**", and "***" represent not significant, P < 0.01, and P < 0.001, respectively.

The clinical evaluations using MG scales indicated significant improvement at each visit (**Figures 4A–C**). Four weeks after the initial dose of ofatumumab, there were significant reductions in the mean values of MG scores: MGFA-QMG decreased by 90.6%, MG-QOL decreased by 87.9%, and MG-ADL decreased by 92.9%. By the 12-week mark, MGFA-QMG, MG-ADL, and MGQOL-15 were further declined compared to baseline, with reductions of 94.7%, 89.3%, and 96.0%, respectively.

Profound depletion of circulating CD19^+^ B was found in MG patients at both 4 weeks (-98.7% compared to baseline, P < 0.001) and 12 weeks (-98.8% compared to baseline, p < 0.001) after initiating ofatumumab therapy (**Figure 4D**). Compared with baseline, AChR antibody levels did not decrease significantly at 4 weeks (P = 0.075) but showed a meaningful reduction after 12 weeks (P=0.001) (**Figure 4E**). Notably, decreases in AChR antibody levels was not correlated with reductions in MG scales at 4 weeks, and it only moderately correlated with improvement in MG-QOL15 (r=0.378, p=0.047) at 12 weeks.

### 3.3 cTfh and cTh17 return to normal after ofatumumab therapy

By the 4-week mark, the frequency of cTfh in MG was significantly lower than at baseline (8.0% vs 9.2%, p = 0.002) but still higher than HCs (8.0% vs 3.3%, p < 0.001) (**Figure 2A**, **Supplementary Figure 2A**). However, 12 weeks after ofatumumab therapy, no statistical difference was observed in the cTfh frequency between MG patients and HCs (3.9% vs 3.3%, p = 0.114) (**Figure 2A**). Similarly, the frequency of cTh17 in MG at 4 weeks exhibited a noticeable decrease compared with baseline (2.9% vs 4.6%, p = 0.008) (**Figure 2B**, **Supplementary Figure 2B**). The frequency of cTh17 showed a further decline in MG at 12 weeks (2.1% at 12 weeks vs 2.1% at 4 weeks, p = 0.012), which was comparable with HCs (2.1% in MG vs 1.7% in HCs, p = 0.446) (**Figure 2B**).

### 3.4 Normalized cTfh and cTh17 associate with clinical improvement

**Figure 5:**
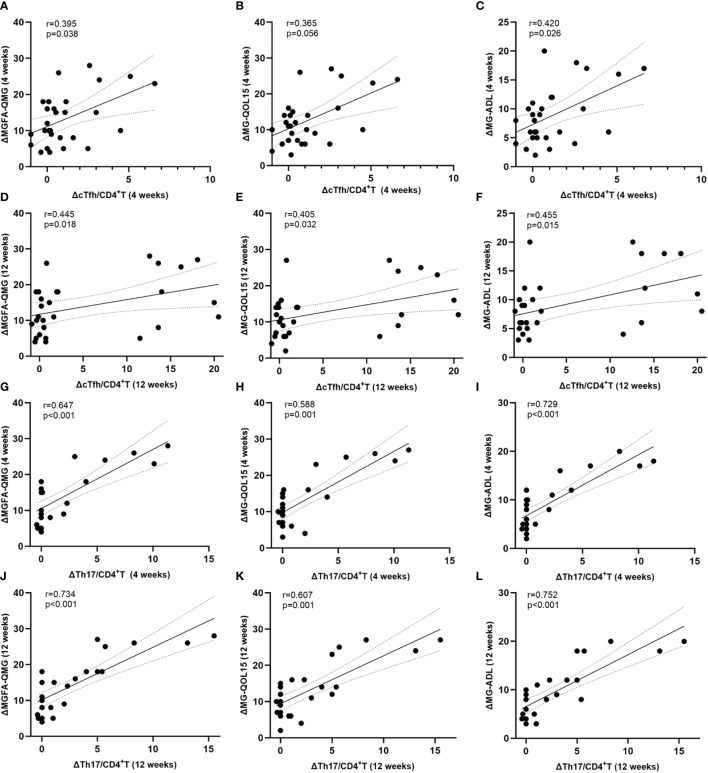
Normalized level of cTfh and cTh17 associate with clinical improvement. **(A–F)** Association between decline in circulating Tfh and Th17 cell frequency and clinical improvement at 4 weeks after initiation of ofatumumab therapy; **(G–L)** Correlation between decreases in circulating Tfh and Th17 cell frequency and clinical mitigation at 12 weeks after initiation of ofatumumab treatment. cTfh, circulating T follicular helper; cTh17, circulating T helper 17; MGFA-QMG, Myasthenia Gravis Foundation of America quantitative myasthenia gravis score; MG-QOL15, the 15-item Myasthenia Gravis Quality of Life scale; MG-ADL, the MG-Related Activities of Daily Living score; ΔMGFA-QMG (4 weeks) = MGFA-QMG_baseline_ - MGFA-QMG_4 weeks_; ΔMG-QOL (4 weeks) = MG-QOL_baseline_ - MG-QOL4 weeks; ΔMG-ADL (4 weeks) = MG-ADL_baseline_ - MG-ADL_4 weeks_; ΔMGFA-QMG (12 weeks) = MGFA-QMG_baseline_ - MGFA-QMG_12 weeks_; ΔMG-QOL (12 weeks) = MG-QOL_baseline_ - MG-QOL_12 weeks_; ΔMG-ADL (12 weeks) = MG-ADL_baseline_ - MG-ADL_12 weeks_; ΔcTfh/CD4^+^T (4 weeks) = cTfh/CD4^+^T_baseline_ - cTfh/CD4 + _4 weeks_; ΔcTfh/CD4^+^T (12 weeks) = cTfh/CD4^+^T_baseline_ - cTfh/CD4 + _12 weeks_; ΔcTh17/CD4^+^T (4 weeks) = cTh17/CD4^+^T_baseline_ - cTh17/CD4 + _4 weeks_; ΔcTh17/CD4^+^T (12 weeks) = cTh17/CD4^+^T_baseline_ - cTh17/CD4 + _12 weeks_.

At 4 weeks after initiating ofatumumab treatment, the decline in the ratio of cTfh was moderately correlated with change in MGFA-QMG (r=0.395, p=0.038) and MG-ADL (r=0.420, p=0.026) but not with MG-QOL15 (r=0.365, p=0.056) (**Figures 5A–C**). At the same time point, there was also a positive correlation between the decline in the ratio of cTh17 and improvement in MG scales (MGFA-QMG: r=0.647, p<0.001; MG-QOL15: r=0.588, p=0.001; MG-ADL: r=0.729, p<0.001) (**Figures 5D–F**). At 12 weeks, correlation analysis revealed that the alternation of cTfh frequency was positively correlated with improvement in clinical scores (MGFA-QMG: r=0.445, p=0.018; MG-QOL15: r=0.405, p=0.032; MG-ADL: r=0.455, p=0.015) (**Figures 5G–I**). The similar phenomenon was observed for the change of cTh17 frequency at the same time (MGFA-QMG: r=0.734, p<0.001; MG-QOL15: r=0.607, p=0.001; MG-ADL: r=0.752, p<0.001) (**Figures 5J–L**). Treg possess the ability to suppress immune responses by inhibiting the function of other effector T cells. It is reported that Th17/Treg imbalance is a significant driver in MG progression (17). A positive association was also found between decline in circulating Th17/Treg ratio and clinical improvement after initiation of ofatumumab therapy (**Supplementary Figure 1**).

### 3.5 cTfh- and cTh17-related cytokines decreased after ofatumumab therapy

**Figure 6:**
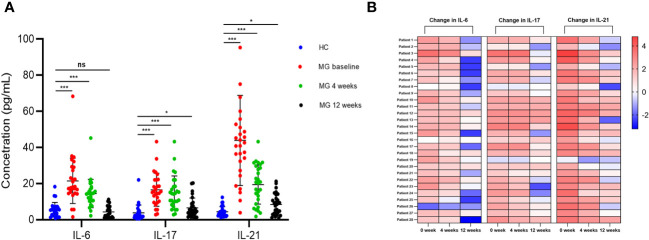
Change of serum IL-6, IL-17, and IL-21 after ofatumumab therapy. **(A)** Comparison of serum levels of IL-6, IL-17 and IL-21 in MG and HC patients at each visit; **(B)** The heatmap demonstrates the relative changes in serum cytokine levels for each individual with MG, relative to the average cytokine levels observed in the HC group. Each column represents a specific cytokine at every visit, while each row corresponds to an MG patient. The varying intensity of color within each square on the heatmap represents the extent of fold change in cytokine levels. MG, myasthenia gravis; HC, healthy control. "ns", "*", and "***" represent not significant, P < 0.05, and P < 0.001, respectively.

BCDT is associated with a reduction in the production of IL-6, which plays a role in the differentiation of Th17 and Tfh cells (18). Additionally, IL-17 and IL-21 are primarily produced by activated Th17 and Tfh cells, respectively (19, 20). Therefore, we examined the levels of these three cytokines in the serum of patients with MG and HCs. At baseline, there were noticeable differences in the serum levels of IL-6 (21.5 pg/mL vs 5.6 pg/mL, p < 0.001), IL-17 (16.5 pg/mL vs 3.8 pg/mL, p < 0.001), and IL-21 (43.9 pg/mL vs 4.4 pg/mL, p < 0.001) between MG patients and HCs (**Figures 6A, B**). However, at the 4-week follow-up, the fold difference in the levels of IL-6 (14.5 pg/mL vs 5.6 pg/mL, p < 0.001), IL-17 (14.9 pg/mL vs 3.8 pg/mL, p < 0.001), and IL-21 (19.5 pg/mL vs 4.4 pg/mL, p < 0.001) between MG patients and HCs were minimized (**Figures 6A, B**). By the 12-week mark, the difference in the levels of the IL-17 (6.5 pg/mL vs 3.8 pg/mL, p = 0.010), IL-21 (8.4 pg/mL vs 4.4 pg/mL, p = 0.010) in MG patients and HCs were further reduced (**Figures 6A, B**). At the same time point, no difference was found in the levels of IL-6 between MG patients and HCs (4.3 pg/mL vs 5.6 pg/mL, p = 0.198) (**Figures 6A, B**).

## 4 Discussion

In our study, two key points were identified: (1) Patients with MG exhibited abnormal expansion of cTfh and cTh17 cells. Their levels returned to normal after ofatumumab therapy; (2) The restoration of cTfh and cTh17 to normal levels was more associated with clinical improvement in this patient population. Therefore, cTfh and cTh17 cells are potential biomarkers for therapeutic efficacy in MG receiving BCDT.

The quantity of cTh17 cells is increased in thymoma-associated MG. It is positively correlated with the MGFA-QMG score and anti-AChR antibody titer (21). It has been proposed that Th17 cells affect autoantibody production by influencing the balance of Th1- and Th2-related cytokines in MG patients (22). In addition, the frequency of cTfh was significantly elevated in patients with MG compared with HC (6, 23). Functionally activated cTfh are key features in the immunopathology of MG and was correlated with disease severity (6) and levels of serum anti-AChR antibody (23). Our study also found similar results to those described above (**Figures 2A, B**, **3A–F**). Furthermore, we demonstrated that decreases in cTfh and cTh17 cell percentages were more associated with clinical improvement in MG than the change in serum anti-AchR antibody titer. Taken together, the expansion of cTfh and cTh17 may contribute to the immunopathogenesis of MG.

Interestingly, 12 weeks after initiating ofatumumab therapy, MG patients showed a significant decrease in the frequencies of cTh17 and cTfh cells in our research. This supports the theory that anti-CD20 agents deplete B cells that have potential crosstalk with T cell subsets (10, 24, 25). A significant decrease in the frequency of cTfh cells has been observed in multiple sclerosis patients following BCDT treatment, and this reduction has been associated with disease remission (26). These observations suggest that BCDTs may indirectly deplete potentially pathogenic cTfh cells, highlighting their interdependence with B cells. In addition, ex vivo experiments (about NMOSD) showed that both IL-6 and direct B/cTfh cell contact were essential for the maintenance of cTfh cells. BCDT inhibited the expansion of cTfh cells through ablating IL-6- secreting B cells and blocking direct contact (25). To be noted, IL-6 also plays a role in the differentiation of Th17 and Tfh cells (18). Consistently, our findings demonstrated that BCDT resulted in a significant reduction in the serum IL-6, IL-17, and IL-21, with the latter two cytokines being primarily generated by Th17 and Tfh cells.

However, we cannot entirely rule out the possibility that the decrease in cTfh and cTh17 cells was due to the direct clearance effect of the ofatumumab. It is worth noting that, in addition to B cells, there exists a small subset of T cells that express CD20 on their cell surface (27). This is supported by recent research, where ofatumumab demonstrated an effective reduction of peripheral CD20^+^ T cells in multiple sclerosis (28). Furthermore, in rheumatoid arthritis patients, it has been observed that a higher proportion of cTh17 cells exhibit a CD20^+^ phenotype compared to healthy controls (29). Overall, these observations suggest that the effects of ofatumumab on both B cells and CD20-expressing T cells should be taken into consideration when interpreting the changes in cTfh and cTh17 cell percentages in our study. Further research is warranted to better understand the specific mechanisms underlying these observations and their implications for clinical outcomes.

This study has several limitations. First, we focused on studying changes in circulating Tfh and Th17 cells in MG patients after BCDT. However, we were unable to detect Tfh cells in lymphoid tissue germinal centers because these cells are difficult to obtain in the routine clinical setting. Second, we did not assess the expression of CD20 markers on cTfh and cTh17 cells. As mentioned previously, it is possible that BCDT depletes specific subpopulations of cTfh and cTh17 cells by binding directly to their surfaces. Third, ficoll isolation was shown to decrease the expression of specific chemokine receptors (e.g., CCR5 and CXCR5) on PBMCs (30, 31). By contrast, staining cells at 37°C following ficoll separation or staining whole blood prior to lysing red blood cells may minimized artifacts on the immunophenotyping (31). Thus, in our study, standard ficoll gradient density separation may have compromised the accuracy of identification of cTfh cells. Fourth, some patients had a short corticosteroid withdrawal period before starting BCDT treatment. As corticosteroids can reduce the proportion of circulating Tfh cells (32), we cannot fully eliminate their confounding effect on T-cell phenotype. Finally, this study had a small sample size and a relatively short follow-up period. Large-scale, long-term studies are needed to confirm the value of cTfh and cTh17 cells in monitoring BCDT efficiency in MG.

## 5 Conclusion

Abnormal expansion of cTfh and cTh17 cells may be key features in the immunopathology of MG. Their levels returned to normal after BCDT, which was closely correlated with clinical amelioration. This result suggests that these two T-cell subsets may be targets for BCDT treatment of MG.

## Data availability statement

The raw data supporting the conclusions of this article will be made available by the authors, without undue reservation.

## Ethics statement

The studies involving humans were approved by The Ethics Committee of Peking University People’s Hospital. The studies were conducted in accordance with the local legislation and institutional requirements. The participants provided their written informed consent to participate in this study.

## Author contributions

XS: Conceptualization, Formal analysis, Investigation, Methodology, Writing – original draft. YHe: Data curation, Investigation, Supervision, Writing – review & editing. YHu: Writing – review & editing. HJ: Data curation, Investigation, Supervision, Writing – review & editing. YY: Data curation, Investigation, Writing – review & editing. YS: Data curation, Investigation, Supervision, Writing – review & editing. ZL: Conceptualization, Formal analysis, Investigation, Methodology, Supervision, Writing – review & editing. ZZ: Conceptualization, Formal analysis, Funding acquisition, Investigation, Resources, Supervision, Writing – review & editing.

## Supplementary material

Supplementary Figure 1 Normalized level of circulating Th17/Treg ratio associate with clinical improvement. **(A)** Reprehensive gating strategy for circulating Treg cell; **(B-D)** Association between decline in circulating Th17/Treg ratio and clinical improvement at 4 weeks after initiation of ofatumumab therapy; **(E-G)** Correlation between decreases in circulating Th17/Treg ratio and clinical mitigation at 12 weeks after initiation of ofatumumab treatment. cTh17, circulating T helper 17; Treg, T regulatory; MGFA-QMG, Myasthenia Gravis Foundation of America quantitative myasthenia gravis score; MG-QOL15, the 15-item Myasthenia Gravis Quality of Life scale; MG-ADL, the MG-Related Activities of Daily Living score; ΔMGFA-QMG (4 weeks) = MGFA-QMG_baseline_ - MGFA-QMG_4 weeks_; ΔMG-QOL (4 weeks) = MG-QOL_baseline_ - MG-QOL4 weeks; ΔMG-ADL (4 weeks) = MG-ADL_baseline_ - MG-ADL_4 weeks_; ΔMGFA-QMG (12 weeks) = MGFA-QMG_baseline_ - MGFA-QMG_12 weeks_; ΔMG-QOL (12 weeks) = MG-QOL_baseline_ - MG-QOL_12 weeks_; ΔMG-ADL (12 weeks) = MG-ADL_baseline_ - MG-ADL_12 weeks_; ΔTh17/Treg (4 weeks) = Th17/Treg_baseline_ - Th17/Treg_4 weeks_; ΔTh17/Treg (12 weeks) = Th17/Treg_baseline_ - Th17/Treg_12 weeks_.

Supplementary Table 1 Overview of MG patient characteristics. MG, myasthenia gravis; F, female; M, male; IVIG, intravenous immunoglobulin; MGFA, Myasthenia Gravis Foundation of America; MGFA-PIS, MGFA post-intervention status; PR, pharmacologic remission; MM, minimal manifestations; I, improved.

Supplementary Table 2 Characteristics of MG patients with and without thymoma history. MG, myasthenia gravis; SD, standard deviation; IQR, interquartile range; MGFA-QMG, Myasthenia Gravis Foundation of America quantitative myasthenia gravis score; MG-QOL15, the 15-item Myasthenia Gravis Quality of Life scale; MG-ADL, the MG-Related Activities of Daily Living score; cTfh, circulating T follicular helper; cTh17, circulating T helper 17.
