# Supplementary figures and images for: Normalized circulating Tfh and Th17 associates with improvement in myasthenia gravis treated with ofatumumab
Source: Front Immunol. 2024 Feb 13;15:1280029. doi: 10.3389/fimmu.2024.1280029 (PMC10898244; doi:10.3389/fimmu.2024.1280029)

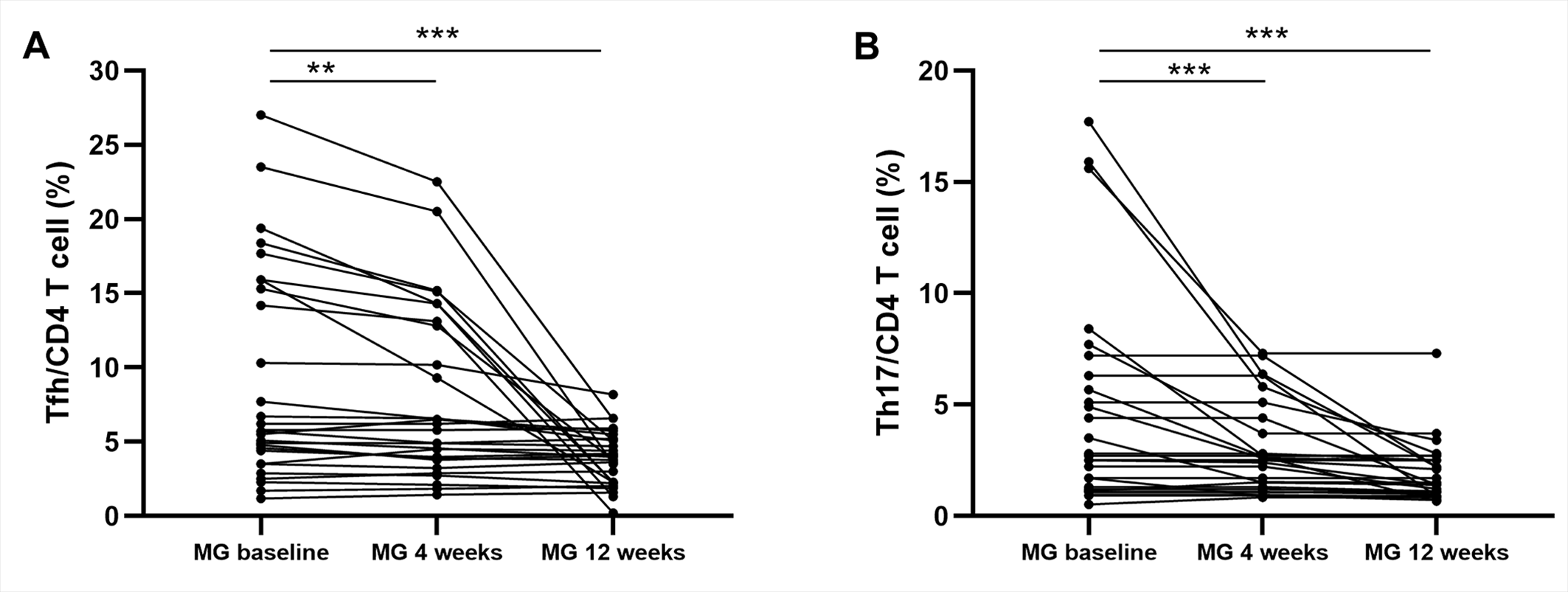

Supplement: Supplementary file 1 [file Image_2.tif]

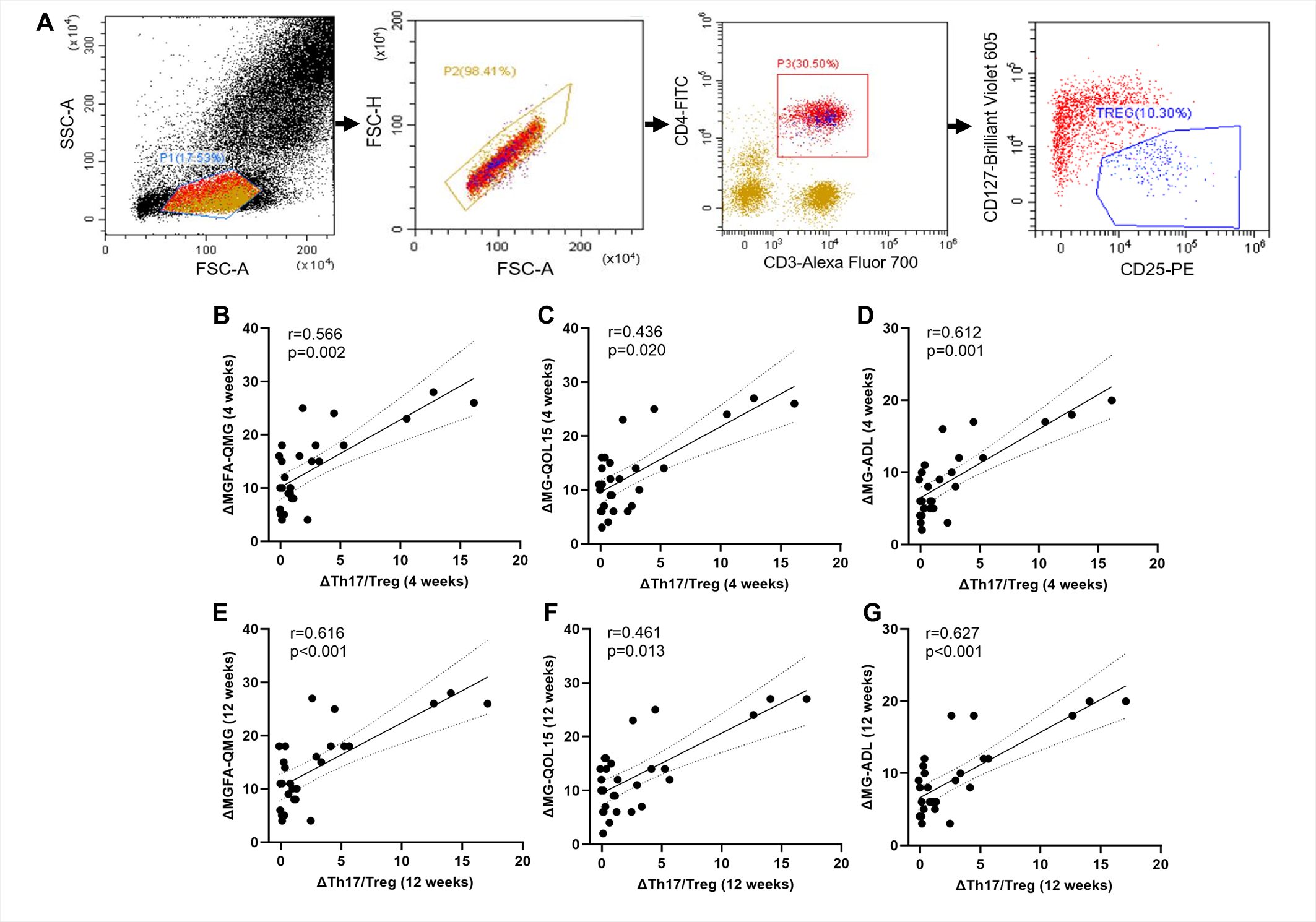

Supplement: Supplementary Figure 1 — Normalized level of circulating Th17/Treg ratio associate with clinical improvement. (A) Reprehensive gating strategy for circulating Treg cell; (B-D) Association between decline in circulating Th17/Treg ratio and clinical improvement at 4 weeks after initiation of ofatumumab therapy; (E-G) Correlation between decreases in circulating Th17/Treg ratio and clinical mitigation at 12 weeks after initiation of ofatumumab treatment. cTh17, circulating T helper 17; Treg, T regulatory; MGFA-QMG, Myasthenia Gravis Foundation of America quantitative myasthenia gravis score; MG-QOL15, the 15-item Myasthenia Gravis Quality of Life scale; MG-ADL, the MG-Related Activities of Daily Living score; ΔMGFA-QMG (4 weeks) = MGFA-QMGbaseline - MGFA-QMG4 weeks; ΔMG-QOL (4 weeks) = MG-QOLbaseline - MG-QOL4 weeks; ΔMG-ADL (4 weeks) = MG-ADLbaseline - MG-ADL4 weeks; ΔMGFA-QMG (12 weeks) = MGFA-QMGbaseline - MGFA-QMG12 weeks; ΔMG-QOL (12 weeks) = MG-QOLbaseline - MG-QOL12 weeks; ΔMG-ADL (12 weeks) = MG-ADLbaseline - MG-ADL12 weeks; ΔTh17/Treg (4 weeks) = Th17/Tregbaseline - Th17/Treg4 weeks; ΔTh17/Treg (12 weeks) = Th17/Tregbaseline - Th17/Treg12 weeks. [file Image_1.tif]
