# Supplementary material for: Normalized circulating Tfh and Th17 associates with improvement in myasthenia gravis treated with ofatumumab
Source: Front Immunol. 2024 Feb 13;15:1280029. doi: 10.3389/fimmu.2024.1280029 (PMC10898244; doi:10.3389/fimmu.2024.1280029)
Supplement: Supplementary Table 1 — Overview of MG patient characteristics. MG, myasthenia gravis; F, female; M, male; IVIG, intravenous immunoglobulin; MGFA, Myasthenia Gravis Foundation of America; MGFA-PIS, MGFA post-intervention status; PR, pharmacologic remission; MM, minimal manifestations; I, improved. [file Table_1.docx]

**Supplementary Table 1 Overview of MG patient characteristics**

| **Patient** | | **Age of onset (years)** | **Sex** | **Disease duration**  **(months)** | **Thymoma type** | **Interval between prior treatment and the initiation of ofatumumab (months)** | **MGFA type** | | **Follow up time after ofatumumab (months)** | **MGFA-PIS at last visit** |
| --- | --- | --- | --- | --- | --- | --- | --- | --- | --- | --- |
| P1 | 62 | M | 18 | B1 | Prednisone (2), thymectomy (12) | | IIIb | 1, 3 ,6, 8, 10 | MM |  |
| P2 | 58 | M | 7 | None | Prednisone (2) | | IIa | 1, 3, 5, 9, 13 | PR |  |
| P3 | 45 | F | 49 | None | Prednisone (3) | | IIa | 1, 3, 7,12 | PR |  |
| P4 | 32 | F | 38 | AB | Prednisone (2), IVIG (6,23,26), thymectomy (17) | | IVb | 1, 3, 6 | MM |  |
| P5 | 75 | M | 26 | None | Prednisone (1.5) | | IIb | 1, 3, 5, 7, 11 | PR |  |
| P6 | 52 | M | 42 | None | Prednisone (1), mycophenolate mofetil (2) | | IIb | 1, 3, 5, 8,12 | PR |  |
| P7 | 53 | F | 18 | B2 | Prednisone (4), thymectomy (3) | | IIIa | 1, 3, 6, 10, 12, 15 | MM |  |
| P8 | 66 | F | 26 | None | Prednisone (2.5) | | IIa | 1, 3, 5, 7, 9 | PR |  |
| P9 | 35 | F | 14 | None | Prednisone (2) | | IIb | 1, 3, 6, 8, 11, 14 | PR |  |
| P10 | 67 | M | 16 | B1 | Prednisone (1), IVIG (6, 12,), thymectomy (12) | | IVb | 1, 3, 6, 11, 12 | I |  |
| P11 | 72 | M | 18 | None | Prednisone (1), IVIG (8) | | IIIb | 1, 3, 6, 10 | PR |  |
| P12 | 63 | M | 16 | None | Prednisone (1), IVIG (10) | | IIIb | 1, 3, 6, 9, 12, 15 | MM |  |
| P13 | 66 | F | 50 | None | Prednisone (2), azathioprine (2) | | IIIa | 1, 3, 11,15 | I |  |
| P14 | 48 | M | 29 | None | Prednisone (2) | | IIb | 1, 3, 5, 8, 11 | PR |  |
| P15 | 55 | M | 19 | AB | Prednisone (1.5), IVIG (7, 14), thymectomy (18) | | IVb | 1, 3, 7 | MM |  |
| P16 | 72 | M | 38 | None | Prednisone (3) | | IIb | 1, 3, 6, 10, 13 | I |  |
| P17 | 65 | M | 24 | None | Prednisone (2), mycophenolate mofetil (2) | | IIIa | 1, 3, 7, 11, 15 | PR |  |
| P18 | 27 | F | 18 | B2 | Thymectomy (12) | | IIa | 1, 3, 7, 11, 13, 16 | PR |  |
| P19 | 68 | M | 12 | B1 | Prednisone (5), IVIG (5), thymectomy (3) | | IIIa | 1, 3, 5, 8, 10, 14 | PR |  |
| P20 | 59 | M | 36 | None | Prednisone (1), tacrolimus (1) | | IIIa | 1, 3, 7, 9, 12 | I |  |
| P21 | 52 | M | 18 | None | Prednisone (2) | | IIa | 1, 3, 6, 8, 11 | PR |  |
| P22 | 33 | F | 4 | None | Prednisone (1.5), IVIG (1,3) | | IVa | 1, 3, 6, 10, 12 | PR |  |
| P23 | 67 | F | 14 | AB | Prednisone (3.5), thymectomy (2) | | IIb | 1, 3, 5, 9, 13 | MM |  |
| P24 | 35 | M | 29 | None | Prednisone (2) | | IIa | 1, 3, 4, 7, 10 | PR |  |
| P25 | 25 | F | 10 | B1 | Prednisone (4), thymectomy (2) | | IIa | 1, 3, 5, 8, 13 | MM |  |
| P26 | 52 | M | 38 | None | Prednisone (1) | | IIb | 1, 3, 5, 9 | PR |  |
| P27 | 44 | F | 7 | None | Prednisone (1) | | IIa | 1, 3, 6, 10 | PR |  |
| P28 | 65 | F | 6 | B2 | Prednisone (3), thymectomy (2) | | IIIa | 1, 3, 5, 7 | PR |  |

*MG: myasthenia gravis; F: female; M: male; IVIG: intravenous immunoglobulin; MGFA: Myasthenia Gravis Foundation of America; MGFA-PIS: MGFA post-intervention status; PR: pharmacologic remission; MM: minimal manifestations; I: improved.*
