# Supplementary material for: Normalized circulating Tfh and Th17 associates with improvement in myasthenia gravis treated with ofatumumab
Source: Front Immunol. 2024 Feb 13;15:1280029. doi: 10.3389/fimmu.2024.1280029 (PMC10898244; doi:10.3389/fimmu.2024.1280029)
Supplement: Supplementary Table 2 — Characteristics of MG patients with and without thymoma history. MG, myasthenia gravis; SD, standard deviation; IQR, interquartile range; MGFA-QMG, Myasthenia Gravis Foundation of America quantitative myasthenia gravis score; MG-QOL15, the 15-item Myasthenia Gravis Quality of Life scale; MG-ADL, the MG-Related Activities of Daily Living score; cTfh, circulating T follicular helper; cTh17, circulating T helper 17. [file Table_2.docx]

**Supplementary Table 2 Characteristics of MG patients with and without thymoma history**

| **Characteristics** | **With thymoma history (n=10)** | **Without thymoma history (n=18)** | **p-value** |
| --- | --- | --- | --- |
| Age (years), mean (SD) | 52.1 (16.5) | 55.1 (12.9) | 0.611 |
| Female, n (%) | 6 (60.0) | 6 (33.3) | 0.172 |
| Disease duration (years), median (IQR) | 17.0 (12.5-18.0) | 26.0 (16.5-37.5) | 0.890 |
| MGFA-QMG score, median (IQR) | 11.5 (6.5-25.0) | 15.0 (10.0-18.0) | 0.724 |
| MG-QOL15 score, median (IQR) | 13.5 (6.3-23.3) | 14.0 (9.8-15.5) | 0.869 |
| MG-ADL score, median (IQR) | 9.5 (5.0-16.5) | 8.0 (6.0-10.0) | 0.906 |
| cTfh/CD4^+^T at baseline (%), median (IQR) | 6.4 (5.1-17.3) | 5.5 (3.1-13.2) | 0.245 |
| cTfh/CD4^+^T at 4 weeks (%), median (IQR) | 6.0 (4.9-13.1) | 5.2 (3.0-12.2) | 0.286 |
| cTfh/CD4^+^T at 12 weeks (%), median (IQR) | 4.6 (3.7-5.2) | 3.7 (2.2-4.9) | 0.286 |
| cTh17/CD4^+^T at baseline (%), median (IQR) | 3.0 (1.8-5.5) | 2.6 (1.2-2.0) | 0.724 |
| cTh17/CD4^+^T at 4 weeks (%), median (IQR) | 2.3 (1.5-2.7) | 2.6 (1.2-4.2) | 0.494 |
| cTh17/CD4^+^T at 12 weeks (%), median (IQR) | 1.5 (1.0-2.0) | 1.9 (1.1-2.7) | 0.265 |

*MG: myasthenia gravis; SD: standard deviation; IQR: interquartile range; MGFA-QMG: Myasthenia Gravis Foundation of America quantitative myasthenia gravis score; MG-QOL15: the 15-item Myasthenia Gravis Quality of Life scale; MG-ADL: the MG-Related Activities of Daily Living score; cTfh: circulating T follicular helper; cTh17: circulating T helper 17.*
